# Plasticized Poly(vinyl chloride) Modified with Developed Fire Retardant System Based on Nanoclay and L-histidinium Dihydrogen Phosphate-Phosphoric Acid

**DOI:** 10.3390/polym13172909

**Published:** 2021-08-29

**Authors:** Kamila Sałasińska, Mateusz Barczewski, Maciej Celiński, Paweł Kozikowski, Rafał Kozera, Aleksandra Sodo, Jacek Mirowski, Stanisław Zajchowski, Jolanta Tomaszewska

**Affiliations:** 1 Faculty of Materials Science and Engineering, Warsaw University of Technology, 05-507 Warsaw, Poland; rafal.kozera@pw.edu.pl (R.K.); ola.sodo@interia.pl (A.S.); 2 Department of Chemical, Biological and Aerosol Hazards, Central Institute for Labour Protection—National Research Institute, 00-701 Warsaw, Poland; maciej.celinski@ciop.pl (M.C.); pawel.kozikowski@ciop.pl (P.K.); 3 Institute of Materials Technology, Poznan University of Technology, 61-138 Poznań, Poland; mateusz.barczewski@put.poznan.pl; 4 Faculty of Chemical Technology and Engineering, UTP University of Science and Technology in Bydgoszcz, 85-326 Bydgoszcz, Poland; jacek.mirowski@utp.edu.pl (J.M.); stanislaw.zajchowski@utp.edu.pl (S.Z.); jolanta.tomaszewska@utp.edu.pl (J.T.)

**Keywords:** burning behavior, flame retardant, thermal stability, dynamic mechanical thermal analysis, cone calorimeter, smoke density chamber, plasticized PVC

## Abstract

The current work assessed the burning behavior of plasticized poly(vinyl chloride) (PVC-P) modified with a two-component composition, consisting of L-histidinium dihydrogen phosphate-phosphoric acid (LHP) and nanoclay (n). The thermal and thermodynamical properties of the PVC-P containing from 10 to 30 wt% of the fire retardant system (FRS) were determined by thermogravimetric analysis (TG) as well as by dynamic mechanical thermal analysis (DMTA). In contrast, fire behavior and smoke emission were studied with a cone calorimeter (CC) and smoke density chamber. The research was complemented by a microstructure analysis, using a scanning electron microscope, of the materials before and after burning CC tests. The effects were compared to those achieved for PVC-P, PVC-P with a commercially available fire retardant, the substrate used for the produced LHP, and the mixture of LHP and zinc borate, both of which contained the same share of nanoclay. Based on a notable improvement, especially in smoke suppression suggests that the n/LHP system may be a candidate fire retardant for decreasing the flammability of PVC-P.

## 1. Introduction

Although the number of fires in the United States decreased by 2.5% from 2009 to 2018, the number of fatalities increased significantly, by as much as 20.5% [1]. Research conducted by the US National Institute of Standards and Technology has shown that the time available for the evacuation of fire victims is only 3 min, while in the 1970s, it was 17 min [2]. The reasons for this change are creating open spaces with fewer barriers (e.g., doors, walls), the presence of larger number of flammable materials, and greater tightness of buildings [3]. These factors contribute to an increase in the intensity of heat release and faster flame spread, an increase in the amount of fumes emitted, and various chemical compounds being present in the fumes [4]. Increases in the toxicity of gases is mainly related to the increase in the share of synthetic materials based on nitrogen and phosphorus, as well as substances containing significant amounts of halogen. Even though the dominant lethal factor is the presence of significant amounts of carbon dioxide, gases such as hydrogen cyanide, hydrogen chloride, hydrogen bromide, and hydrogen fluoride, also cause negative effects on the body, as well as causing confusion and loss of consciousness, which lead to death [5].

Due to its favorable properties, such as a high chemical and environmental resistance, ease of modification, widely developed processing methods, and low cost, poly(vinyl chloride) (PVC) is one of the most commonly used thermoplastics. Moreover, due to its high chlorine content (56.7%), PVC does not burn when the heat source or flame is removed. Currently, European converters’ demand for poly(vinyl chloride) is over 6 million tons per year and is growing steadily, at an average of 5% per year, prompted by increasing investment in construction and infrastructure projects [6]. Due to the presence of stabilizers, fillers, plasticizers, etc., there are two types of poly(vinyl chloride): unplasticized (PVC-U) and plasticized (PVC-P). PVC-P is characterized by high flexibility, transparency, and ease of processing, as well as resistance to weather conditions. Additionally, it is impermeable to odors and oxygen, but degrades at elevated temperatures [7]. PVC-P products primarily include boards and foils, cables, extruded profiles, artificial leather, floor coverings, and textiles [8].

Fire safety is an essential issue in poly(vinyl chloride) production, especially in products used in the furniture, electrical, electronic, construction, and transportation industries. Poly(vinyl chloride) burns with a yellow flame, and the harsh smell of volatile combustion products results from hydrogen chloride release. It was determined that when poly(vinyl chloride) is heated in an anaerobic atmosphere at temperatures of 227–277 °C, the destruction of the polymer and the release of gaseous hydrogen chloride takes place. The next step is carbonization, which occurs at a temperature of 427–480 °C and is accompanied by hydrogen evolution. This process involves intramolecular reactions in which poly(vinyl chloride) is converted into cyclic compounds (mainly benzene), and the reactions lead to the cross-linking of non-volatile decomposition products. The temperatures at which the process takes place depends on the amount of stabilizers added [9].

A way to reduce its flammability is through the introduction of fire retardant additives. Among these additives, the solutions described below deserve particular attention. US Patent No. US5886072 designated flame retardant compositions containing aluminum hydroxide, diantimony trioxide, brominated phthalic esters, isodecyl diphenyl phosphate, ammonium octamolybdate, and zinc borate for use in polyvinyl sheaths for electric cables. The mentioned mixtures allow increases to the value of the oxygen index of poly(vinyl chloride) of up to 48%. The international patent EP 2875069B1 described flame-retardant PVC compositions containing flame retardant agents, e.g., diantimony trioxide, zinc borate, and aluminum and calcium hypophosphites. The investigated compositions obtained the V-0 class in the UL-94 flammability test. In turn, US Patent No. US3962177 disclosed a series of mixtures of nickel and aluminum compounds to reduce poly(vinyl chloride) smoke formation. The best result was obtained from a mixture of nickel oxalate and zinc-aluminium sulphate, which allowed reductions in the amount of smoke released by 75%. Tai et al. [10] used organically modified iron-montmorillonite (Fe-OMT) and zinc borate (ZnB) to improve the flame retardancy of glass fibre reinforced polyamide 6 (GFPA6), containing melamine polyphosphate (MPP). It was shown that a composite modified with 28 wt% of MPP and 2 wt% of either ZnB or Fe-OMT could achieve UL-94 V0 rating, exhibiting better flame retardancy than GFPA6 with 30 wt% of MPP.

The subject of the present invention is a two-component composition that reduces the smoke emission of polymers during the burning process, consisting of L-histidinium dihydrogen phosphate-phosphoric acid (LHP) and nanoclay (n). The content of the composition in the plasticized PVC ranges from 10 to 30% by weight. Research methods, including thermogravimetric analysis, cone calorimetry, and smoke density chamber measurements, were employed to determine the impact of FRS on the polymer’s thermal stability and burning behavior. Moreover, the effect of the addition of FRS on the mechanical and thermomechanical properties was determined using a tensile test and a dynamic-mechanical non-isothermal analysis carried out under the pre-determined conditions of mechanical excitation. The investigation was complemented by a microstructure analysis of the samples before and after burning measurements were taken. The results were compared to those obtained for PVC-P, as well as for PVC-P with a commercially available fire retardant, histidine as the substrate used for the produced LHP, and the mixture of LHP and zinc borate, which contained the same share of nanoclay.

## 2. Materials and Methods

The compounds used in the synthesis of LHP were histidine (H) from Apollo Scientific (Stockport, UK) and phosphoric acid from Honeywell (Charlotte, NC, USA). The composition was a dry blend of poly(vinyl chloride) which included: PVC S61 Neralit 601 from Spolana Ltd. (Neratovice, Czech RepublicPW) (100 phr), plasticizer DINP from Boryszew S.A. (Warsaw, Poland) (30 phr), thermal stabilizer Patstab 2310 from Patcham Ltd. (Saif Zone, Sharjah, UAE) (3 phr), and wax Naftolube FTP from Chemson Inc. (Arnoldstein, Austria) (1 phr). Commercial fire retardants, such as ammonium polyphosphate (APP) from Walter Thieme Handel GmbH (Stade, Germany) and zinc borate (ZB) from Nordmann Rassmann GmbH (Hamburg, Germany), were applied. In turn, the nanoclay Nanomer^®^ I.44P (n), modified by silane with mean particle sizes below 20 μm, was obtained from Sigma-Aldrich Co (Darmstadt, Germany).

**Figure 1:**
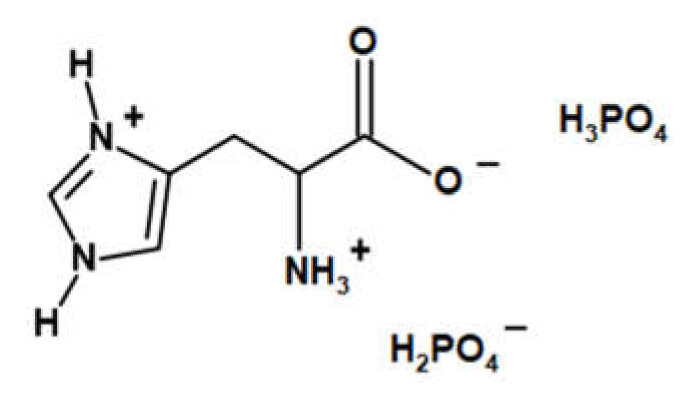
The chemical structure of LHP [12].

LHP acid (Figure 1) was synthesized according to the previously reported procedure [11,12].

The PVC dry blend was prepared using a low-speed mixer for 30 min with a rotor speed of 30 rpm and a chamber temperature of 90 °C [13]. Next, the PVC dry blend was mixed with fire retardant additives at a temperature of 20 °C by means of a high-speed mixer of own construction. The PVC compounds with various type and amount of FRs were processed by extrusion using a co-rotating twin-screw extruder EHP 2 × 24 M with L/D = 40 (Zamak Mercator, Skawina, Poland). The temperatures of the following cylinder zones were: 100, 110, 115, 120, 130, 140, 150, 155, and 160 °C, and the head temperature was 150 °C. In the last stage, the gelated compounds were ground and the samples were produced with the use of a hydraulic press PHM-63 (Ponar Żywiec, Żywiec, Poland). The temperature, pressure, and duration of the processes were 180 °C, 12 MPa, and 8 ± 1 min, respectively [13]. The compositions of the mixtures and the designations of samples are given in Table 1.

A scanning electron microscope SU8010 (Hitachi, Tokio, Japan) was used to study the materials’ morphology before and after burning tests. The specimens for the SEM observations were gold-coated by employing a Quorum Technologies Q150T ES sputter coater (Quorum Technologies, Laughton, UK). At the same time, the residues did not require sputter coating and were fixed on a table using LEIT-C Conductive Carbon Cement. The observations were carried out at an accelerating voltage of 5 ÷ 10 kV and magnifications of 1000×. The point elemental analysis was performed using the Thermo Scientific NORAN System 7 implemented with an electrically cooled Silicon Drift Detector EDS (Thermo Scientific UltraDry, Waltham, MA, USA).

Samples were prepared using an Ultramicrotome Leica EM UC6 (Leica Microsystems, Wetzlar, Germany) in a chamber equipped for low-temperature processing. The PVC was cut into slides with 75 nm thickness with a diamond knife, at a temperature of −40 °C. The samples were transferred into copper meshes with carbon membranes and then observed in bright field mode using a STEM Hitachi S5500 (Hitachi, Tokio, Japan) and 30 kV of accelerating voltage.

The static tensile test was conducted following EN ISO 527, using a Zwick Roell Z010 testing machine (Zwick GmbH & Co., KG, Ulm, Germany). The test specimens (type 5A) were stretched with using a force gauge of 5 kN and extension speed of 1 mm/min to 0.25% strain and 50 mm/min in the later part of the measurement [14].

The dynamic thermomechanical analysis (DMTA) test was performed using an MCR 301 (Anton Paar, Ostfildern-Scharnhausen, Germany) rheometer equipped with a torsion DMA measuring tool. The analyses were conducted at a constant frequency of 1 Hz and a strain of 0.01%. All samples (50 × 10 × 4 mm were cooled down to −45 °C and heated up to 90 °C with a temperature ramp of 3 °C/min.

The Vicat softening temperature (VST) was evaluated according to the standard ISO 306, using an RV300C (Testlab, Warsaw, Poland) apparatus with a heating rate of 50 °C/h and a load of 10 N. The VST for each series was determined based on seven measurements.

The thermogravimetric analysis (TGA) method was employed to study the thermal stability of the PVC/FRs in an atmosphere of nitrogen. Samples of 10 ± 0.2 mg were placed in Al_2_O_3_ crucibles and were heated in a temperature range of 30–900 °C, with a 10 °C/min rate, using a TG209 F1 (Netzsch, Selb, Germany) apparatus.

Fire behavior was evaluated by employing a cone calorimeter (Fire Testing Technology Ltd., East Grinstead, UK), following the ISO 5660 standard. The samples, with dimensions of 100 × 100 × 6 mm, were placed in aluminum foil and tested horizontally at a heat flux of 35 kW/m^2^. The separation between the samples and the heater was 25 mm. The spark ignition was used to ignite the pyrolysis products. The residues were then photographed by an EOS 400 D camera (Canon Inc., Tokio, Japan). The values presented here are the averages obtained for three samples from each series. The fire behavior was estimated using the Flame Retardancy Index (FRI), proposed by Vahabi et al. [15], and calculated from the following Equation:

(1) $$\mathrm{FGI}=\frac{\left[\mathrm{THR}*\left(\frac{\mathrm{pHRR}}{\mathrm{TTI}}\right)\right]\mathrm{PVC}}{\left[\mathrm{THR}*\left(\frac{\mathrm{pHRR}}{\mathrm{TTI}}\right)\right]\mathrm{PVC}/\mathrm{FR}}$$

The optical density of the smoke was evaluated using a smoke density chamber (Fire Testing Technology Ltd., East Grinstead, UK) in conformity with the ISO 5659-2 standard. The samples, with dimensions of 75 × 75 × 6 mm, were exposed to a heat flux of 25 kW/m^2^ without applying a pilot flame. The values are the averages obtained for the three samples from each series.

## 3. Results

### 3.1. Microstructure Analysis

**Figure 2:**
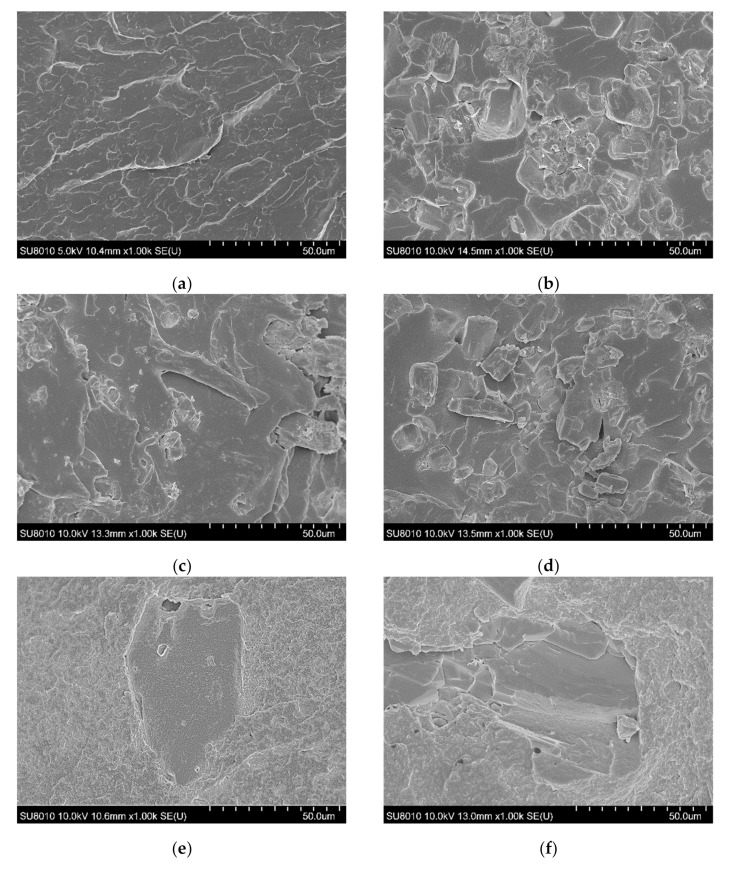

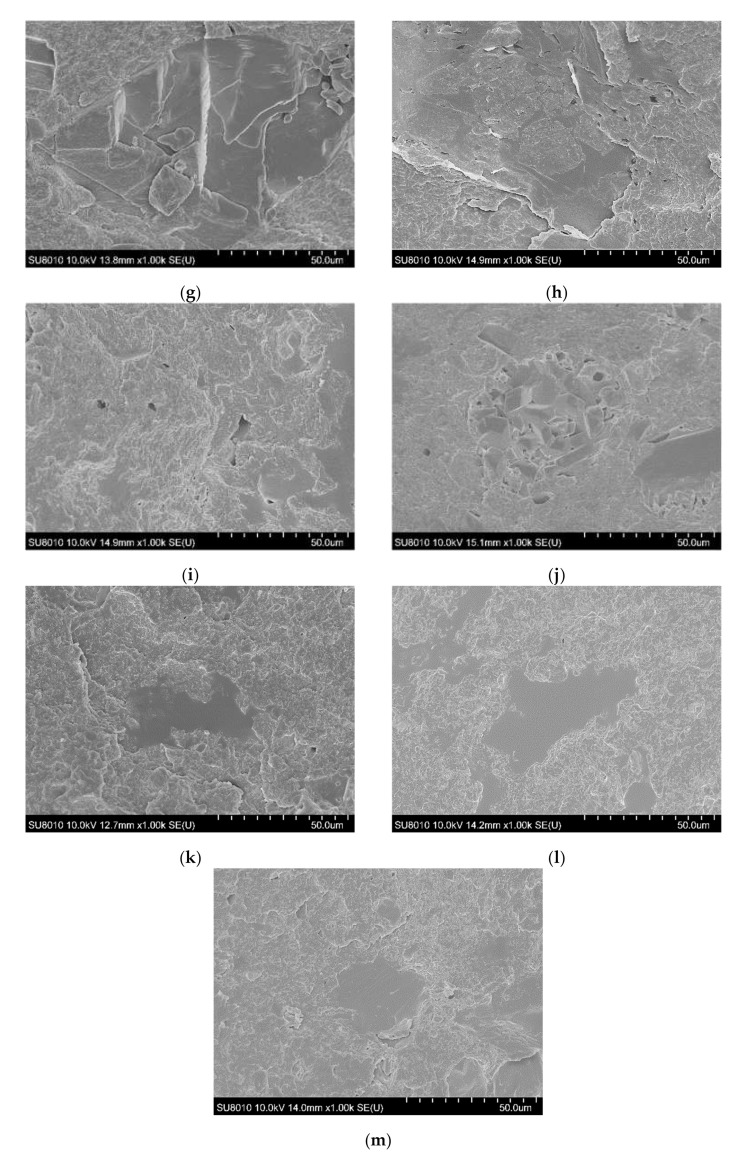
SEM images of fracture surfaces of PVC (**a**), 10APP (**b**), 20APP (**c**), 30APP (**d**), 4n6H (**e**), 4n16H (**f**), 4n26H (**g**), 4n6LHP (**h**), 4n16LHP (**i**), 4n26LHP (**j**), 4n3LHP3ZB (**k**), 4n8LHP8ZB (**l**), and 4n13LHP13ZB (**m**).

The SEM images allowed us to study the morphology of the flexible poly(vinyl chloride) with fire retardant additives, and they are shown in Figure 2.

The PVC-P images (Figure 2a) mostly show numerous parallel deformations due to the rapid spread of energy through the plastic. Moreover, no pores were observed in the material. An analysis of the poly(vinyl chloride) microstructure with APP showed relatively uniformly distributed particles, however, a few agglomerates were also observed. Pores related to the loss of fire retardant particles indicate poor adhesion between APP and the polymer. Figure 2e–g shows images of the poly(vinyl chloride) microstructure with the histidine and nanoclay. The histidine particles were of considerable size and distributed throughout the volume of the material. A large number of free pores in the polymer were also observed. In turn, the analysis of PVC-P with LHP and nanoclay demonstrated notably sized particles, but also their agglomerates and pores (Figure 2j). The uniform areas, visible especially for PVC-P with LHP, ZB, and n (Figure 2l), may suggest that the swelling process started prematurely. This is consistent with the presence of large pore sizes in the microstructure of the n/LHP/ZB samples. In most series, the progress in defects with the increase in the share of FRs was observed. The observation of nanoclay particles and their agglomerates could not be achieved.

**Figure 3:**
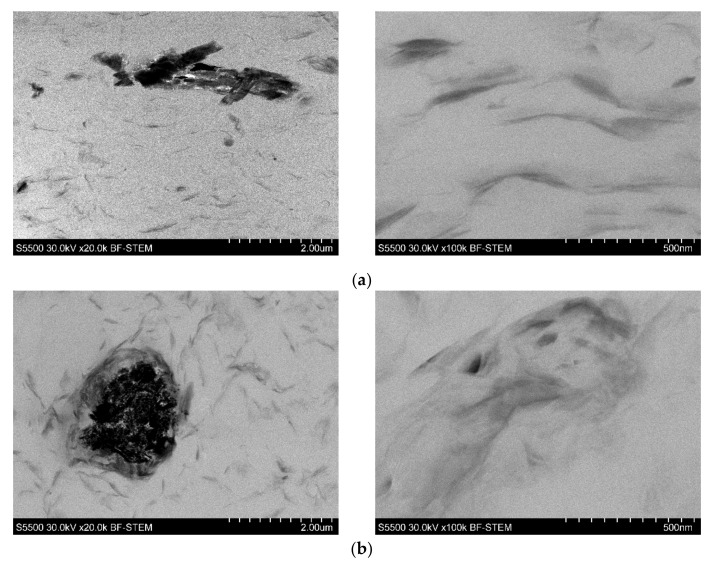

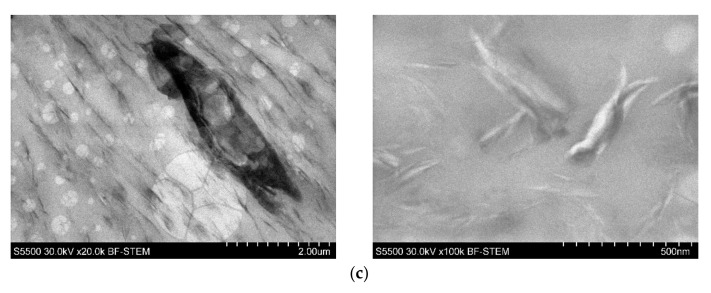
TEM images of 4n6H (**a**), 4n6LHP (**b**), and 4n3LHP3ZB (**c**).

For this reason, the morphological studies were supplemented by the TEM investigation of the selected samples. The images presented in Figure 3 reveal excellent dispersion of the nanoclay in the PVC-P. However, the agglomerates of the FR’s particles, as well as pores, can also be observed. As can be seen, apart from intercalated structures, the morphology was comprised of exfoliated structures.

### 3.2. Mechanical Performance

**Figure 4:**
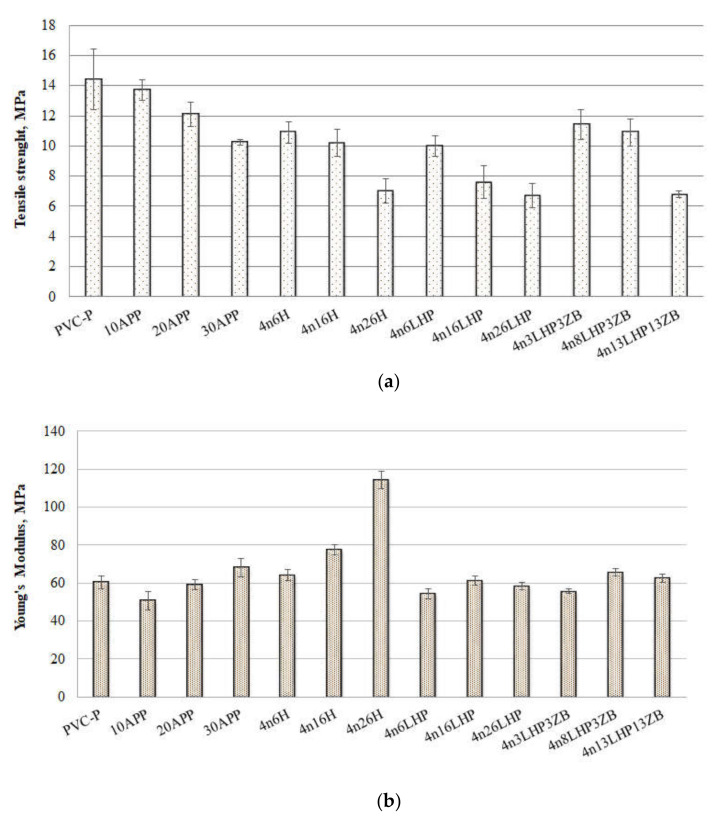
Tensile strength (**a**) and Young’s modulus (**b**) results of PVC/FRs obtained from tensile tests.

Tensile strength tests were conducted to assess the influence of the investigated fire retardant system on the mechanical properties of plasticized PVC, and the results are presented in Figure 4.

Mechanical properties, except for the shape of particles, may relate to their distribution and interactions between the additive and the polymer [16]. The introduction of fire retardants caused a reduction in tensile strength for all of the materials (Figure 4a), and this parameter was further reduced as the amount of additives increased. This is probably due to the stress concentration around the particles, which may be attributed to insufficient adhesion between the components [17,18,19] and is convergent with our previous research [13]. The poor adhesion of components in APP samples resulted in a reduction in tensile strength value from 5 to 29%, depending on the FR share. Additionally, the presence of free pores reduced it by a further 24–51% for the n/H series, while the uneven distribution, pores, and agglomerates in n/LHP materials resulted in depression of tensile strength by 31–53%. The addition of 10 wt% fire retardants caused a decrease in the Young’s modulus (E). This relationship did not apply to histidine-modified materials, which is probably an effect of its significant particle size and uniform distribution. As the amount of additives increased, an increase in the elastic modulus of the materials was observed. The largest change, an almost two-fold increase in comparison to the unmodified thermoplastic, was obtained for 4n26H. The E values closest to PVC were obtained for the n/LHP and n/LHP/ZB series. Either refraining from influencing this property or enacting only a small increase will allow the application of the PVC/FRs in similar areas as the plasticized PVC.

**Figure 5:**
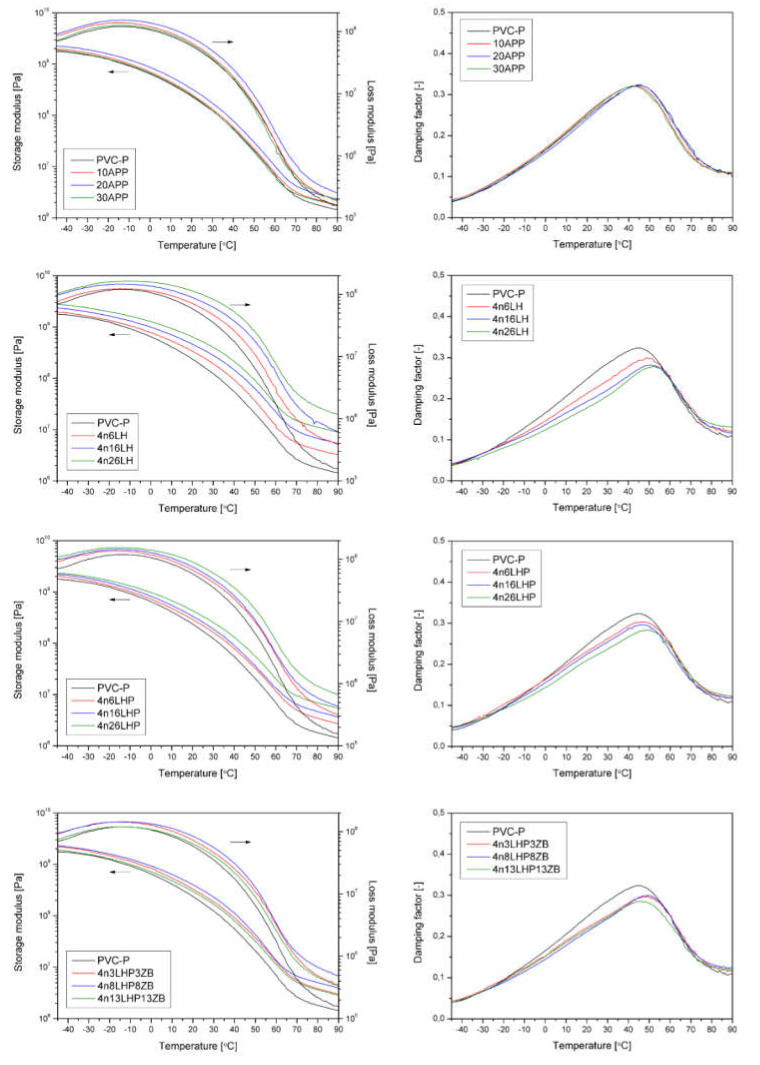
Storage and loss moduli and damping factors vs. temperature curves of pure and modified PVC-P.

The mechanical properties’ evaluation was completed by dynamic-mechanical tests carried out under the determined conditions of mechanical excitation. Figure 5 shows the results of the DMA, presented as a storage (G′) loss (G″) moduli and damping factor (tanδ) as a function of temperature, recorded for PVC-P and modified with different FRs. Additional thermomechanical data, including the storage modulus determined at −40, 25, and 80 °C, as well as the maximum value of G″ and the glass transition temperature (Tg) determined as the peak of tanδ(T) curve [20], are presented in Table 2. All modified compositions showed improved stiffness, interpreted as a higher storage modulus, in comparison to the reference PVC-P sample. The greater the amount of FR, the higher the value of G′. The most distinct modifying effect was noted for PVC-P with the addition of histidine. The 4n26H revealed a G′ almost five times higher in a room and at an elevated temperature (Table 2), resulting from the significant particle size. For the APP and n/LHP/ZB-modified series, another tendency was noted. The highest stiffness of the materials was observed for 20 wt% of the filler. Further increases in FR content resulted in a lower storage modulus across the entire temperature range considered. This may be related to the poor adhesion and porosity of both modifying systems in the plasticized PVC-P. Despite a different deformation system, the DMA results align with the results of the mechanical properties assessed in the static tensile tests. The trend of G′ changes corresponds to the observed changes in Young’s modulus. Only in the n/H series case were significant changes in stiffness noted, which translated into improved G′ compared to unmodified PVC-P across the entire range.

The analysis of relaxation temperatures may be a sensitive tool for evaluating the compatibility of PVC-P with various additives [21]. The maximum of the tanδ curve has been used as a criterion for determining Tg, according to Daniels and Cabrera [20]. Considering the results of the structural changes in the modified PVC-P obtained from the SEM analysis, the reduction in Tg observed for the APP-modified series cannot be equated with the modification of the plasticizing effect, but rather with the resulting structural defects, both caused by improper adhesion at the interface. The tanδ value is straightly connected with changes in polymer chain mobility.. The increased maximum of tanδ vs. the T curve may suggest the creation of additional interactions between macromolecules and additives and/or reinforcing the effect of the modifier used [22]. Therefore, the increase in Tg for PVC-P modified H and LHP with nanoclay, and considering the simultaneous increase in G′, can be interpreted as a system achieving high compatibility [23].

Vicat softening temperature assessment is one of the most frequently used industrial thermomechanical parameters for evaluating thermoplastics’ application range. Moreover, it can also be a valuable tool for supplementing information about structural changes and the miscibility of polymeric compositions [24,25,26]. Referring to previous literature studies, the introduction of insoluble and inert additives to plasticized PVC, including flame retardants and fillers, may induce different tendencies (both increases and decreases) in PVC-P the thermomechanical stability as assessed by the VST criterion [27,28,29]. The results of the VST measurements are presented in Table 2. The addition of all FRs caused an increase in the Vicat softening temperature compared to the reference PVC-P sample. The effectiveness of the incorporated PVC-P modifiers was comparable to the case of 10 wt%. Further increases in the FRs content caused VST diversification, and the most favorable changes were noted for the sample containing the highest concentration of histidine (69.2 °C), while the lowest was in the series containing LHP (63.1 °C). However, it should be emphasized that in the case of all modified PVC-P series, even those with a minor amount of the modifier, an increase in the VST of approximately 10 °C was recorded, which translates into a significant increase in the scope of their applicability. The results achieved in our experiments are comparable to those observed by Petersen et al. [28], who reported the effects of organo-modified montmorillonite (OMMT) interactions on the thermal properties of PVC, and noted the most favorable effect in the case of a 5% nanofiller introduced into PVC plasticized by means of a 40 wt% tributyl citrate.

### 3.3. Thermal Stability

**Figure 6:**
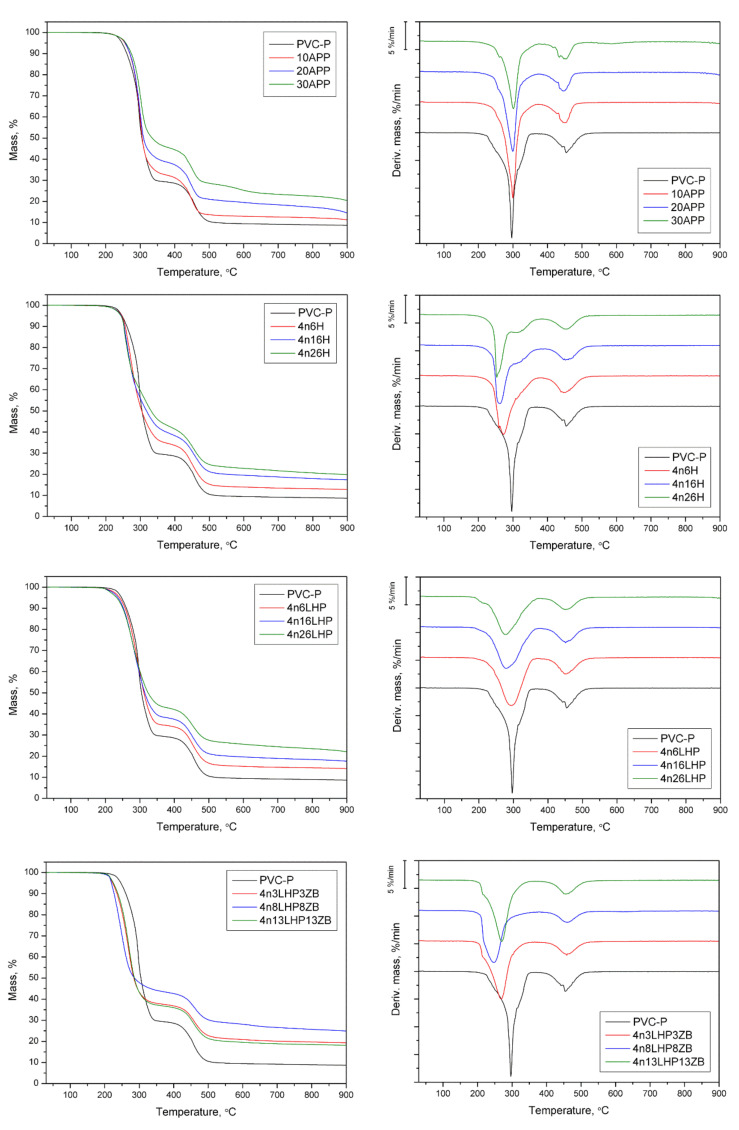
TG and DTG curves of PVC-P, PVC/APP, and PVC/FRSs under an inert atmosphere.

The thermal stability and degradation behavior of PVC-P with FR systems were studied via thermogravimetric analysis. Based on the obtained mass vs. temperature curves, its first derivative (DTG) was calculated. Values of 5% mass loss (T_5%_), maximum thermal degradation intensity (DTG peak), the rate of decomposition, and residual mass were determined. The details of the decomposition process are summarized in Table 3, while the TG and DTG curves are presented in Figure 6.

The thermal degradation of unmodified poly(vinyl chloride) was characterized by two main decomposition steps, read from DTG with the maximum mass loss occurring at 297 °C and 454 °C, respectively, and the residue after test was approx. 8.7% (Figure 6). The addition of fire retardants had a significant effect on the initial thermal stability, temperature, rate of peaks, and especially on residue yields (Table 3). PVC-P’s initial degradation temperature (T_5%_) reached 247 °C, while the commercial FR improved T_5%_, and the values grew slightly as the amount of APP increased. The opposite trend was noted for thermoplastic with developed FR systems, for which a gradual decrease in T_5%_, or a rapid decline as seen in n/LHP/ZB, was observed. Similar variations were noted for the first peak (1st DTG peak), while there was no clear relationship in the case of the second peak (2st DTG peak). Yang et al. [30] also observed a related phenomenon in the thermal decomposition of PBT/clay nanocomposites. It was stated that the catalytic locations in nanoclay layers, including absorbed water, modifiers, and the activity hydroxyl groups, might accelerate the thermal degradation of the polymer. Generally, the decomposition process of PVC includes two main steps, corresponding to the dehydrochlorination of PVC and the degradation of formed conjugated polyene sequences, leading to the formation of char. In the prime decomposition step of the investigated PVC-P, apart from dehydrochlorination, (which includes the autocatalysis process by releasing HCl), oxidation of the plasticizer occurred [31,32]. The incorporation of FRSs, which are multi-component systems, substantially altered the decomposition of PVC-P. In the first stage, the loss of water from the nanoclay, as well as from zinc borate (2 ZnO • 3 B_2_O_3_ • 3.5 H_2_O) in the case of the n/LHP/ZB series, occurred. Additionally, the zinc from ZB catalyzes the release of halogens through the formation of zinc halides and zinc oxyhalides. Liu et al. [31] demonstrated that the addition of Sb-LDH could accelerate the dehydrochlorination of PVC by absorbing the hydrogen chloride during heating, and its release at the first step. In turn, during the thermal degradation of H and LHP from developed FRSs, as the result of the breakdown of the amino group, gaseous ammonium and nitrogen oxides form, while the acidic group decomposes into water and carbon oxides favorable to char formation. The histidine may also undergo the inner cyclization process, forming a 2-amino-2,4-cyclopentadiene-1-one and imidazole [33]. Furthermore, phosphoric acid from dehydrates form either phosphorus pentoxide or polyphosphoric acid, which co-create the char structure in the case of the LHP series [11].

The addition of APP caused an increase in the rate of degradation, notably in the 1st step, and the values decreased inversely to the yield of residue. In the n/H and n/LHP/ZB series, only slight increases in the 1st step and decreases in the 2nd step were observed. In turn, significant decreases in both peaks of n/LHP were observed. The result is attributed to the slowing of the release of volatiles due to the formation of thermally stable products that remained in the residue [30]. Consequently, the slower decomposition rate was due to the carbonaceous char’s formation, evident in the developed fire retardant systems. The lowest values were recorded for samples with LHP, corresponding to the highest yields of residue (above 20%). The lower residue yield of 4n13LHP13ZB suggests an uneven distribution of FRS particles in the material, the effect of which is particularly evident in tests carried out with small samples.

In summary, the combination of phosphorus and nitrogen from the LHP with nanoclay slowed down the mass loss rate during the degradation, both in the first and second degradation steps, leading to improvements in char formation.

### 3.4. Forced Flaming Fire Behavior (Cone Calorimeter)

**Figure 7:**
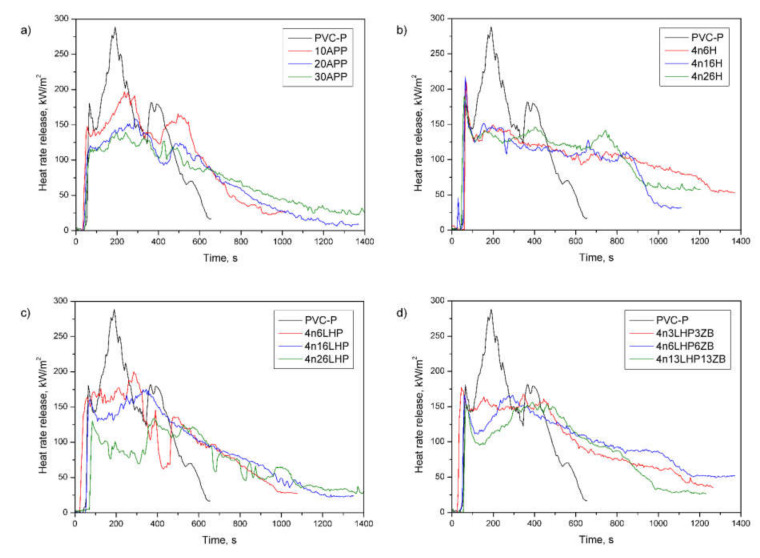
Representative curves of heat release rates of the PVC-P and PVC-P with APP (**a**) or n/H (**b**), n/LHP (**c**), or n/LHP/ZB (**d**) systems.

**Figure 8:**
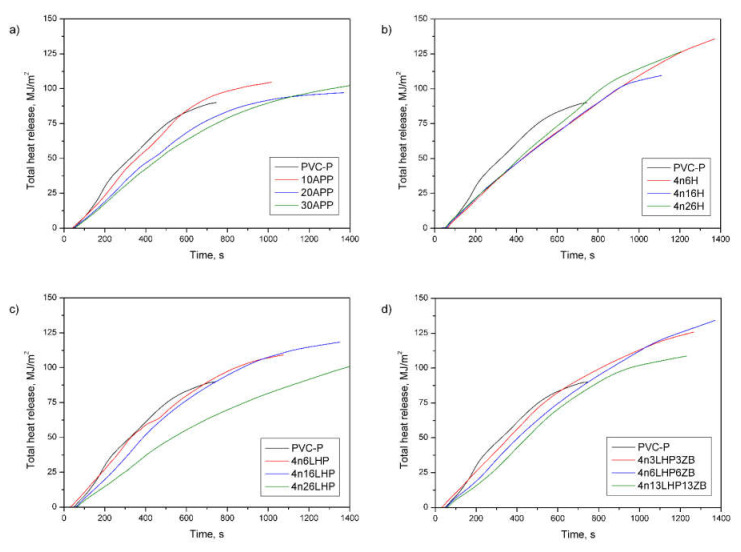
Representative curves of total heat release of the PVC-P and PVC-P with APP (**a**) or n/H (**b**), n/LHP (**c**), or n/LHP/ZB (**d**) systems.

Cone calorimeter measurements simulate a developing fire scenario using a small sample and are fundamental to characterizing the fire retardancy behavior of polymers. Figure 7 and Figure 8 display the heat release rate (HRR) and the total heat release (THR) over time, while the values of the rest of the basic parameters of the test, such as the time to ignition (TTI), the peak heat release rate (pHRR), and the maximum average rate of heat emission (MAHRE) are shown in Table 4.

The HRR curve of the PVC-P exhibit that the material volatilized and ignited in only tens of seconds, reaching the maximum value (peak HRR) at approx. 200 s from the test started. As shown in Figure 7, there are three peaks, which result from the formation of successive char and a temporary reduction in the heat release rate. However, the volatile decomposition products deformed the mechanically weak protective layers and disrupted its continuity, which was confirmed by an openwork structure at the top of the samples, shown in Figure 8a. In turn, the HRR curves of flame-retarded materials flattened, which is characteristic of thick-charring specimens. The dehydration process in plastics provokes cyclization and cross-linking, following by aromatization and graphitization [34], which, combined with the release of non-flammable gases, develops a swelled char layer at the specimen’s surface. Furthermore, the presence of the nanoclay promotes the continuity of the layer, and zinc borate, simultaneously supporting the formation of char, creates a protective layer of glass. Therefore, the pHRR for flame-retarded samples are lower compared to the non-flame retarded one, where the pHRR reached 282 kW/m^2^ (Table 4). However, additional peaks caused by the cracking of the protective layer can also be observed [35,36]. The lowest pHRR (138 kW/m^2^) was noted in the case of 4n26LHP, for which the highest time to ignition (TTI) was also observed. In general, ignition in cone calorimetry measurements occurs when the mass loss rate allows the release of sufficient volatiles to make a gas mixture capable of being ignited by a spark, and occurs if the material’s surface temperature equals its ignition temperature [35]. Interestingly, the addition of LHP caused a considerable reduction in TTI, but the values increased as the amount of FR grew. The same was trend observed for the n/LHP and n/LHP/ZB series, but the opposite results were recorded in samples with histidine. This may be related to the microstructure of the samples as well as to the ratio of nanoclay to other components of the fire retardant system, and its ability to transfer heat.

To assess the hazard of developing fires, some indicates deduced from the maximum HRR, such as the maximum average rate of heat emission (MARHE), are applied. Similar to the pHRR, the values decreased as the FR content increased, and the lowest one was obtained for 4n26LHP (reduction by 45%). Additionally, the Flame Retardancy Index (FRI), used for quantifying the flame retardancy of the thermoplastics based on data from cone calorimetry (TTI, pHRR, THR), was employed. Based on the research conducted by Vahabi et al. [28], most of the flame retarded samples exhibited an FRI ranging from 0 to 10, wherein the values below 0 were not satisfactory (the lower limit for flame retardancy) and those above 10 are labeled as excellent. The FRI values were from 0.6 to 2.2, and the highest result was noted for PVC-P with 30 wt% of n/LHP.

Apart from the flame spread or HRR, the fire load is considered to have the most noticeable impact on the fire hazard [35]. As shown in Table 4, all fire retardants cause an increase in the total heat release (THR), and these values are independent of the share of additives. Figure 8 displays the THR, which is the total heat output versus time. From the curves’ profile, it can be observed that fire retarded PVC-P samples did not reach higher THR values compared to the unmodified polymer during the first 600 s, but they burned much longer than the PVC-P, which may have affected the results. Moreover, the effective heat of combustion of the volatiles, which is an indicator for gas-phase activity [35,37], also increased. Similarly, the EHC is neither affected by the type nor the amount of FR used, since most results had a comparable range of values. The addition of the FRSs of interest caused the change in the gas-phase activity by increasing the char yield, which was confirmed by a decrease in mass loss (Table 4) and an increase in the amount of Cl element in the residue of the n/LHP/ZB material (Figure 10). In samples with a constant effective heat of combustion, the THR is controlled by the mass loss, corresponding to the difference between the initial and the residual mass [35].

**Figure 9:**
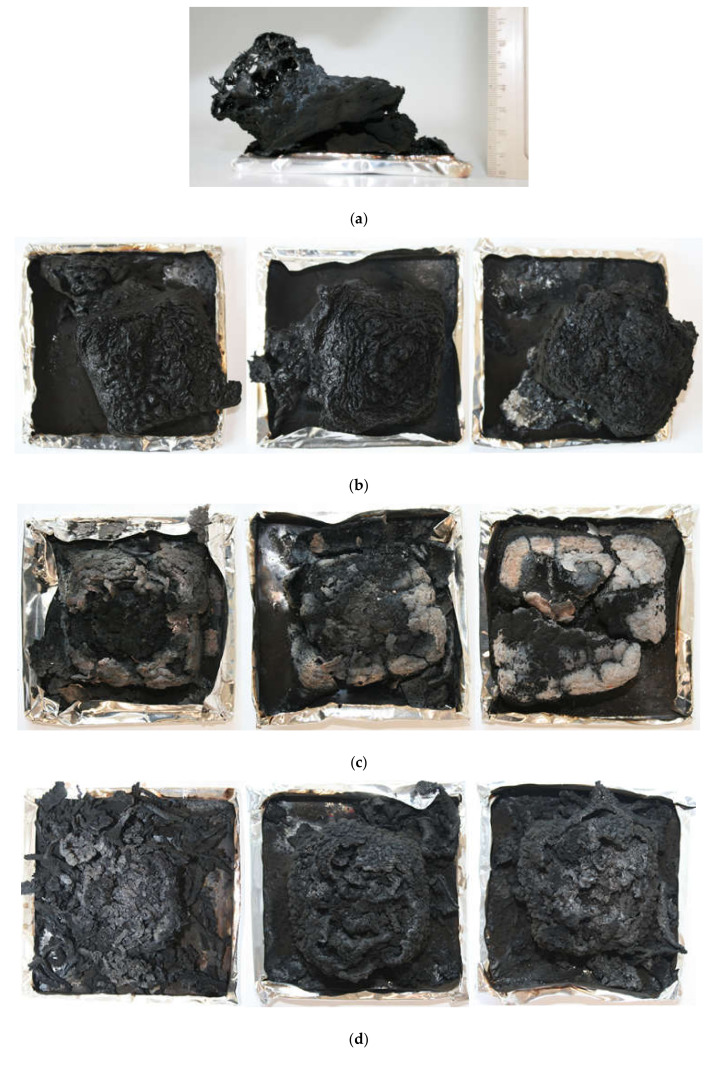

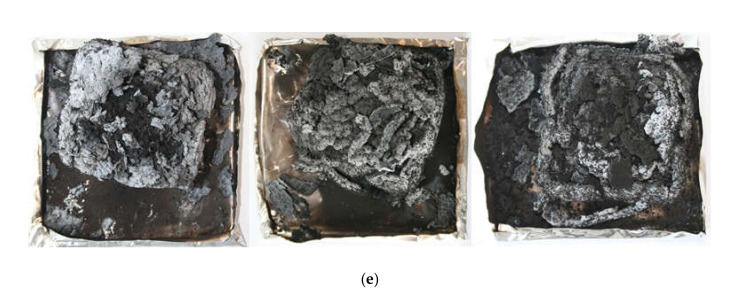
Photographs of the PVC-P (**a**), 10-30 APP (**b**), 10-30 n/H (**c**), 10-30 n/LHP (**d**), and 10-30 n/LHP/ZB (**e**) after cone calorimetry tests.

The residue is regulated by the sample mass as well as by the char yield [35]. Figure 9 presents the samples obtained after cone calorimeter measurements. PVC-P formed swollen char without a continuous outer layer, and apart from the considerable size, it covered only part of the mould. The residues that covered the largest area were formed by PVC-P with different amounts of histidine. Moreover, the samples containing APP and H maintained their shape after burning, whereas the rest became more brittle and broke easily. This means that for samples with H, the nanoclay addition allowed the material to retain its shape. Bearing in mind the application of flexible poly(vinyl chloride), shape retention increases the safety of the material. However, the most swollen char structures were observed for the n/LHP and n/LHP/ZB series. The swollen and tough char, if of an appropriate size and containing inflammable gases inside the pores, can prevent heat and oxygen transfer and consequently prohibit the evolution of flammable polymer decomposition products into the combustion zone, thus improving the flame retardancy of the material.

**Figure 10:**
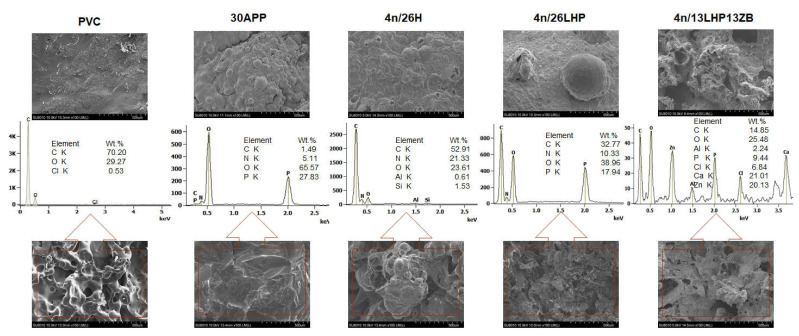
SEM images of PVC-P and PVC-P with APP or n/H, n/LHP, n/LHP/ZB systems after CC tests and EDS results (outer part of a char—top images and inner part of char—bottom images).

A swollen structure, visible in the inner part of the residues in Figure 10, proves that developed fire retardant systems act as intumescent fire retardants. The chemical composition revealed that the residues were comprised mainly of C and O, but also P, N and Cl elements or Zn in the case of n/LHP/ZB. Ca and Zn in the case of n/LHP/ZB derived from nanoclay and zinc borate decomposition products, respectively. In turn, Si elements detected for some samples probably were the contamination, while the Al derived from the sample’s aluminium cover.

### 3.5. Smoke Emission

Flexible poly(vinyl chloride) is widely used in housing and public utility buildings. However, in a fire it produces dense smoke, which hinders escape due to poor visibility, and increases health risks through smoke inhalation. It is worth emphasizing that both smoke and toxic CO production result from incomplete combustion. Most fire retardants work through flame inhibition, resulting in remarkably increased yields of smoke and toxic fumes [34].

**Figure 11:**
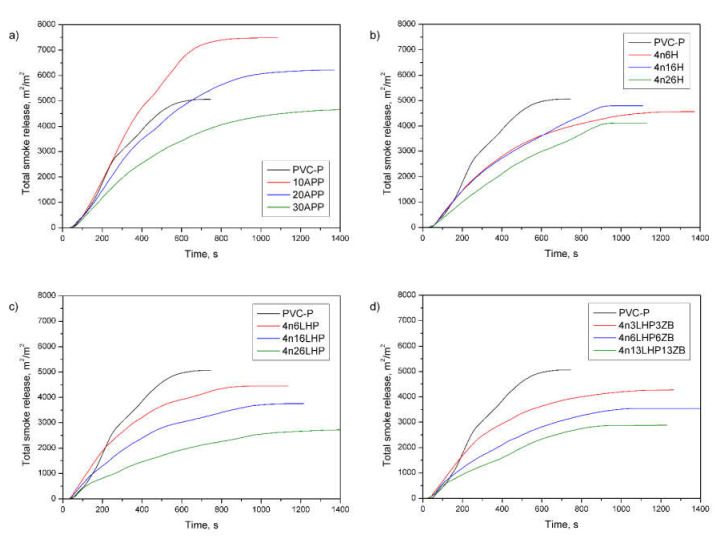
Representative curves of total smoke release of the PVC-P and PVC-P with APP (**a**) or n/H (**b**), n/LHP (**c**), and n/LHP/ZB (**d**) systems.

The total smoke release (TSR), obtained from the forced flaming combustion of a cone calorimeter test, designates the cumulative smoke amount generated per unit area of the sample [34,36]. From the curves’ profile (Figure 11), it is observed that most of the samples exhibited a significant decrease in TSR during the entire measurement compared to the unmodified polymer and commercial FR. Notably, the amount of emitted smoke successively decreased as the share of developed fire retardants increased (Table 4). The lowest value noted for 4n26LHP was 2674 m^2^/m^2^, representing a 47% and 42% reduction compared to PVC-P and 30APP, respectively. Comparable effects were reported for a specific extinction area (SEA), matching the surface of light-absorbing particles present in the smoke generated from 1 kg of material. A SEA of 4n26LHP (351 m^2^/kg) was 48% lower than both the samples of PVC-P and 30APP, definitive evidence of the strong smoke suppression effects of the FR system being investigated.

Additionally, the smoke density chamber tests were performed in order to assess whether or not the smoke suppressant mode in confined spaces corresponded with the prior premise. The highest maximum specific optical density (D_Smax_) was recorded for PVC-P. Similar to the above results, along with the increased FRs share, the D_Smax_ values decreased. The lowest ones were obtained for the n/LHP/ZB series, and the value of 4n13LHP13ZB was about 50% lower than for PVC-P. The suppression of smoke corresponds to the lower mass loss caused by incorporating incomplete burning products into the condensed phase (Table 5). In turn, VOF4, which indicates how much smoke was released during the first 4 min test, increased for almost all samples with LHP.

## 4. Conclusions

This study aimed to investigate the impact of a developed fire-retardant system on plasticized PVC’s burning behavior. The subject of the present investigation was a composition consisting of L-histidinium dihydrogen phosphate-phosphoric acid and nanoclay. The results were related not only to those obtained for PVC-P and PVC-P modified with a commercial fire-retardant but also to histidine, a substrate used to manufacture LHP and LHP and zinc borate mixture, both of which contained the same amount of nanoclay.

The addition of FRSs caused a reduction in the mechanical properties, which may be attributed mainly to the weak adhesion between components and the porosity of the samples. In turn, an increase in stiffness among materials with histidine was due to their large particle sizes. The PVC-P with developed fire retardant systems were characterized by a lowering of their initial temperature of degradation, but also had a lower decomposition rate and a much higher yield of residue, especially in the case of the n/LHP series.

Under forced-flaming conditions, the material 4n26LHP ignited the slowest compared to the others and had the lowest pHRR and MARHE, while also having the highest FRI. The fire-retardant system under consideration led to an increase in the char yield, confirmed by a decrease in mass loss. The studies of SEM confirmed the swollen structure of carbonaceous char, although only for materials with Hthe samples had a continuous outer layer after burning. Moreover, the improvement in charring significantly affected smoke suppression. The developed FRS acted in the gas phase via flame inhibition and in the condensed phase via char formation.

In summary, the combination of LHP with nanoclay allowed the simultaneous reduction in the intensity of the fire and of smoke emission to values lower than those demonstrated by the popular solutions available on the market. Furthermore, the research carried out for the different compositions of the FRSs confirmed the synergistic effects between the components of this system.

## 5. Patents

Patents’ application “A composition of substances that reduce the smoke emission of polymers during the burning process” No. P.431188.

## Figures and Tables

**Table 1:** Formulations of PVC-P compounds with various types of fire retardants (FRs).

Samples	Components, wt%					
	PVC-P	APP	H	LHP	ZB	n
PVC-P	100					
10APP	90	10				
20APP	80	20				
30APP	70	30				
4n6H	90		6			4
4n16H	80		16			4
4n26H	70		26			4
4n6LHP	90			6		4
4n16LHP	80			16		4
4n26LHP	70			26		4
4n3LHP3ZB	90			3	3	4
4n8LHP8ZB	80			8	8	4
4n13LHP13ZB	70			13	13	4

**Table 2:** Thermomechanical properties of pure and modified PVC-P.

Samples	G′_−40 °C_	G′_25 °C_	G′_80 °C_	T at G′′ Max	tanδ Peak	Tg at tanδ	VST
	10^9^ Pa	10^8^ Pa	10^6^ Pa	°C	-	°C	°C
PVC-P	1.72	1.70	1.79	−13.7	0.324	45.4	51.5 ± 0.7
10APP	1.92	1.83	2.25	−14.7	0.321	43.0	58.8 ± 0.6
20APP	2.18	2.40	2.92	−13.2	0.322	44.8	61.6 ± 0.6
30APP	1.80	1.74	2.18	−13.7	0.321	42.4	67.4 ± 1.2
4n6H	1.87	2.55	3.97	−14.5	0.300	48.7	61.7 ± 1.0
4n16H	2.25	3.60	6.69	−13.9	0.282	49.9	65.5 ± 0.8
4n26H	2.66	5.07	11.1	−10.8	0.278	52.3	69.2 ± 0.7
4n6LHP	1.93	2.16	3.43	−16.4	0.303	46.5	59.3 ± 0.5
4n16LHP	2.12	2.52	4.62	−15.5	0.296	46.4	60.4 ± 0.9
4n26LHP	2.25	3.31	6.83	−15.2	0.283	49.2	63.1 ± 0.6
4n3LHP3ZB	2.11	2.47	3.80	−16.2	0.299	49.1	61.0 ± 1.2
4n8LHP8ZB	2.21	3.03	4.99	−13.5	0.300	49.3	62.2 ± 0.6
4n13LHP13ZB	1.80	2.22	3.62	−13.5	0.284	46.5	65.7 ± 0.6

**Table 3:** TG and DTG data of unmodified PVC-P and thermoplastic with APP or FR systems.

Samples	T_5%_	1st DTG Peak	2nd DTG Peak	Residual Mass
	°C	°C; %/min		%
PVC-P	247	297; −9.02	454; −3.61	8.7
10APP	255	300; −17.29	450; −3.72	11.3
20APP	257	300; −14.39	447; −3.50	14.5
30APP	258	302; −12.17	454; −3.21	20.4
4n6H	247	273; −10.60	449; −3.06	12.8
4n16H	246	263; −10.54	452; −2.69	17.4
4n26H	244	253; −11.33	454; −2.59	19.9
4n6LHP	242	294; −8.67	445 −3.00	14.1
4n16LHP	237	278; −7.48	451; −2.86	17.6
4n26LHP	233	228; −6.87	451; −2.35	22.0
4n3LHP3ZB	223	269; −10.46	459; −2.58	19.3
4n8LHP8ZB	219	249; −9.39	456; −2.16	24.0
4n13LHP13ZB	223	267; −10.69	456; −2.53	18.8

**Table 4:** Cone calorimeter results of PVC-P and PVC/FR.

Samples	TTI, s	pHRR, kW/m^2^	MARHE, kW/m^2^	FRI, -	THR, MJ/m^2^	EHC, MJ/kg	Mass Loss, %
PVC-P	60 ± 20	282 ± 10	163 ± 5	-	90 ± 5	12.4 ± 1	87 ± 3
10APP	40 ± 10	206 ± 9	141 ± 2	0.8	108 ± 5	14.0 ± 1	86 ± 1
20APP	56 ± 7	161 ± 7	113 ± 4	1.5	97 ± 10	14.0 ± 2	79 ± 1
30APP	47 ± 2	144 ± 8	109 ± 7	1.3	142 ± 31	15.6 ± 1	70 ± 1
4n6H	60 ± 2	204 ± 11	118 ± 8	0.9	114 ± 7	18.2 ± 4	88 ± 1
4n16H	51 ± 19	179 ± 35	117 ± 8	1.1	122 ± 4	15.3 ± 1	84 ± 1
4n26H	47 ± 17	166 ± 22	122 ± 4	1.0	112 ± 4	16.7 ± 1	79 ± 1
4n6LHP	30 ± 2	185 ± 21	147 ± 10	0.6	112 ± 8	14.2 ± 1	88 ± 1
4n16LHP	50 ± 10	170 ± 6	122 ± 7	1.1	101 ± 9	14.6 ± 1	83 ± 0
4n26LHP	71 ± 5	138 ± 3	89 ± 8	2.2	120 ± 6	14.1 ± 1	76 ± 0
4n3LHP3ZB	34 ± 2	181 ± 24	136 ± 6	0.7	142 ± 30	15.8 ± 1	84 ± 1
4n8LHP8ZB	50 ± 15	171 ± 15	124 ± 10	0.9	109 ± 1	19.3 ± 4	78 ± 2
4n13LHP13ZB	60 ± 3	163 ± 10	122 ± 6	1.4	142 ± 31	15.8 ± 0	72 ± 0

**Table 5:** Cone calorimeter results of PVC-P and PVC/FR.

Samples	TSR, m^2^/ m^2^	SEA, m^2^/kg	Ds_max_	VOF4
PVC-P	5034 ± 150	680 ± 13	1209 ± 134	434 ± 31
10APP	7481 ± 273	965 ± 32	1028 ± 3	375 ± 50
20APP	6160 ± 205	850 ± 13	954 ± 31	239 ± 38
30APP	4613 ± 128	684 ± 7	918 ± 0	291 ± 42
4n6H	4704 ± 302	592 ± 27	906 ± 65	462 ± 106
4n16H	4899 ± 13	646 ± 18	851 ± 38	296 ± 68
4n26H	4289 ± 156	578 ± 22	889 ± 38	295 ± 15
4n6LHP	4417 ± 140	558 ± 12	961 ± 32	609 ± 72
4n16LHP	3878 ± 291	499 ± 33	878 ± 59	474 ± 58
4n26LHP	2674 ± 140	351 ± 23	776 ± 33	373 ± 79
4n3LHP3ZB	4336 ± 273	569 ± 28	924 ± 33	533 ± 118
4n8LHP8ZB	3573 ± 285	469 ± 42	717 ± 40	265 ± 330
4n13LHP13ZB	2999 ± 169	411 ± 25	678 ± 2	480 ± 5

## Data Availability

The data presented in this study are available on request from the corresponding author.
